# Comparison of the validity of smear and culture conversion as a prognostic marker of treatment outcome in patients with multidrug-resistant tuberculosis

**DOI:** 10.1371/journal.pone.0197880

**Published:** 2018-05-23

**Authors:** Kefyalew Addis Alene, Kerri Viney, Hengzhong Yi, Emma S. McBryde, Kunyun Yang, Liqiong Bai, Darren J. Gray, Zuhui Xu, Archie C. A. Clements

**Affiliations:** 1 Research School of Population Health, College of Health and Medicine, The Australian National University, Canberra, Australian Capital Territory, Australia; 2 Institute of Public Health, College of Medicine and Health Sciences, University of Gondar, Gondar, Ethiopia; 3 Department of Public Health Sciences, Karolinska Institutet, Stockholm, Sweden; 4 Department of MDR-TB, Internal Medicine, Hunan Chest hospital, Changsha city, Hunan Province, China; 5 Australian Institute of Tropical Health and Medicine, James Cook University, Townsville, Queensland, Australia; 6 Department of Director's Office, Tuberculosis Control Institute of Hunan Province, Changsha city, Hunan Province, China; 7 Department of Tuberculosis Control, Tuberculosis Control Institute of Hunan Province, Changsha city, Hunan Province, China; Indian Institute of Technology Delhi, INDIA

## Abstract

**Background:**

The World Health Organization (WHO) has conditionally recommended the use of sputum smear microscopy and culture examination for the monitoring of multidrug-resistant tuberculosis (MDR-TB) treatment. We aimed to assess and compare the validity of smear and culture conversion at different time points during treatment for MDR-TB, as a prognostic marker for end-of-treatment outcomes.

**Methods:**

We undertook a retrospective observational cohort study using data obtained from Hunan Chest Hospital, China and Gondar University Hospital, Ethiopia. The sensitivity and specificity of culture and sputum smear conversion for predicting treatment outcomes were analysed using a random-effects generalized linear mixed model.

**Results:**

A total of 429 bacteriologically confirmed MDR-TB patients with a culture and smear positive result were included. Overall, 345 (80%) patients had a successful treatment outcome, and 84 (20%) patients had poor treatment outcomes. The sensitivity of smear and culture conversion to predict a successful treatment outcome were: 77.9% and 68.9% at 2 months after starting treatment (difference between tests, p = 0.007); 95.9% and 92.7% at 4 months (p = 0.06); 97.4% and 96.2% at 6 months (p = 0.386); and 99.4% and 98.9% at 12 months (p = 0.412), respectively. The specificity of smear and culture non-conversion to predict a poor treatment outcome were: 41.6% and 60.7% at 2 months (p = 0.012); 23.8% and 48.8% at 4 months (p<0.001); and 20.2% and 42.8% at 6 months (p<0.001); and 15.4% and 32.1% (p<0.001) at 12 months, respectively. The sensitivity of culture and smear conversion increased as the month of conversion increased but at the cost of decreased specificity. The optimum time points after conversion to provide the best prognostic marker of a successful treatment outcome were between two and four months after treatment commencement for smear, and between four and six months for culture. The common optimum time point for smear and culture conversion was four months. At this time point, culture conversion (AU_ROC_ curve = 0.71) was significantly better than smear conversion (AU_ROC_ curve = 0.6) in predicting successful treatment outcomes (p < 0.001). However, the validity of smear conversion (AU_ROC_ curve = 0.7) was equivalent to culture conversion (AU_ROC_ curve = 0.71) in predicting treatment outcomes when demographic and clinical factors were included in the model. The positive and negative predictive values for smear conversion were: 57.3% and 65.7% at two months, 55.7% and 85.4% at four months, and 55.0% and 88.6% at six months; and for culture conversions it was: 63.7% and 66.2% at two months, 64.4% and 87.1% at four months, and 62.7% and 91.9% at six months, respectively.

**Conclusions:**

The validity of smear conversion is significantly lower than culture conversion in predicting MDR-TB treatment outcomes. We support the WHO recommendation of using both smear and culture examination rather than smear alone for the monitoring of MDR-TB patients for a better prediction of successful treatment outcomes. The optimum time points to predict a future successful treatment outcome were between two and four months after treatment commencement for sputum smear conversion and between four and six months for culture conversion. The common optimum times for culture and smear conversion together was four months.

## Introduction

Multidrug-resistant tuberculosis (MDR-TB) is a public health crisis that is a threat to tuberculosis (TB) control programs in several parts of the world [1]. The number of patients being enrolled onto MDR-TB treatment has been increasing globally, from 45,881 in 2010 to 125,000 in 2015 [2, 3]. Treatment for MDR-TB takes from nine to 24 months and the response to MDR-TB treatment is often slow [4]. An outcome of treatment is assigned to each patient at the end of each treatment course; these outcomes are usually aligned with recommendations from the World Health Organization (WHO) [5]. Globally, only, half (52%) of the MDR-TB patients who started treatment in 2013 were successfully treated [2]. Patients with MDR-TB are monitored during the course of treatment by monthly laboratory tests (usually by providing sputum, which is used to assess smear and culture status, aiming for conversion to negative for confirmed pulmonary cases). Based on published evidence this is viewed as being the best strategy to evaluate interim and final responses to treatment, determine infectiousness, provide information for clinicians who may be considering a change in or adjustment to the treatment regimen, and is also used to assign final treatment outcomes [6].

Despite major recent developments in TB diagnostic tests, including the introduction of an automated molecular test, MDR-TB treatment response is still evaluated using microbiological techniques such as sputum smear examination and culture [7, 8]. The new WHO MDR-TB treatment guideline, released in October 2016, recommends the use of sputum smear microscopy and culture rather than sputum smear microscopy alone for the monitoring of patients with MDR-TB during treatment [5]. This recommendation has been described as a conditional recommendation with very low quality evidence; and it will be revised based on future available evidence [5].

Sputum culture is resource intensive, requires specialised laboratories, equipment and trained staff, takes time to obtain a result and is costly [9, 10]. Therefore, it is not readily available in resource constrained settings, or if it is, it may only be available at reference laboratories, at referral hospitals or in capital cities [9, 11]. On the other hand, sputum smear microscopy is relatively inexpensive, provides rapid results, is easy to perform, does not require complex laboratory equipment and is therefore very suitable for peripheral health centres and low-resource settings [9, 12–14]. However, the validity (i.e. the sensitivity, specificity) and the predictive value (i.e. the positive and negative predictive value) of sputum smear conversion (from positive to negative) at different time points has not been systematically studied as a proxy marker for a successful MDR-TB treatment outcome, as compared to culture conversion. In addition, the optimum times of both culture and smear conversion to predict a final successful TB treatment outcome is not well known. There are few studies that have examined the validity of culture conversion at different time-points to predict end of treatment outcomes [15–17]. The studies that have been identified report that culture conversion at six months is better than culture conversion at two months in predicting successful MDR-TB treatment outcomes [16, 17]. A limitation of the current studies is that they have not assessed and compared the validity of culture and smear conversion as prognostic markers of MDR-TB treatment outcomes at different time-points. Thus, we used data from two high MDR-TB burden countries, China and Ethiopia, to assess and to compare the validity and predictive value of culture and smear conversion status at different time points as prognostic markers for a successful MDR-TB treatment outcome.

## Methods

### Study design and settings

A retrospective observational cohort study was conducted using data obtained from Hunan Chest Hospital, China and Gondar University Hospital, Ethiopia. Hunan Chest Hospital is located in Changsha, the capital city of Hunan province, and Gondar University Hospital is located in Northwest Ethiopia. The Hunan Chest Hospital and the Gondar University Hospital established MDR-TB treatment centers in 2011 and 2010, respectively. These hospitals provide comprehensive diagnostic and treatment services for all persons with presumptive drug resistant TB who live in their catchment area. As part of routine care, sputum smear microscopy and culture are performed monthly for the first six months of treatment, and thereafter every second month until the end of the treatment. At Gondar University Hospital, sputum specimens are sent to the national or regional laboratory for culture; whereas at the Hunan Chest Hospital, solid and liquid culture is performed in the hospital’s laboratory. The treatment regimens and the diagnostic procedures of MDR-TB patients in Hunan Chest Hospital and in Gondar University Hospital have been described in detail elsewhere [18, 19]. Smear microscopy with Ziehl-Neelsen staining and fluorescence microscopy are used in both Hunan Chest Hospital and Gondar University Hospital (1, 2) for both the diagnosis and monitoring of TB. Since culture is not available at Gondar University Hospital, sputum specimens are sent to the national or regional laboratories for culture and drug susceptibility testing (DST). Solid (Löwenstein-Jensen) and liquid culture (BACTEC 460, MGIT 960) are performed at the national and regional laboratories. All MDR-TB patients included in our study were diagnosed and monitored using solid culture. Whereas, at the Hunan Chest Hospital, liquid (BACTEC 460, MGIT 960) and solid cultures (Löwenstein-Jensen) are performed in the hospital’s laboratory. In our study, almost all cases (98%) from Honan Chest Hospital were diagnosed and monitored using solid culture.

### Inclusion and exclusion criteria

This study included all MDR-TB patients who were registered at both Hospitals since the establishment of their MDR-TB treatment centres through until 31^st^ of December, 2014. This period was purposely selected in order to obtain treatment outcomes of the patients (as treatment is 24 months in duration). All bacteriologically confirmed MDR-TB patients (i.e. sputum smear and culture positive) at the commencement of the treatment were eligible. Patients who were diagnosed with MDR-TB but who did not start treatment were excluded. In addition, patients who were lost to follow-up and patients who were diagnosed with XDR-TB were excluded.

### Definitions

We referred to WHO guidelines when defining smear and culture conversion and treatment outcomes [7, 8]. We defined culture conversion as two consecutive negative sputum cultures taken at least 30 days apart following an initial positive culture [7]. Similarly, we defined smear conversion as two consecutive negative sputum smears taken at least 30 days apart following an initial positive sputum smear [7]. We defined time to initial sputum smear conversion as the time in months from the date of start of MDR-TB treatment to the date of specimen collection for the first of two consecutive negative sputum smear results, irrespective of whether there was a subsequent sputum smear positive result. We defined smear reversion to positive when at least one subsequent positive sputum smear was recorded after initial conversion. We defined sustained sputum smear conversion as an absence of any subsequent positive sputum smear after conversion. We also defined persistent sputum smear positivity as no sputum smear conversion in patients with a baseline positive sputum smear. We used similar definitions for time to initial culture conversion, culture reversion and persistent culture positivity (S1Table).

All confirmed MDR-TB patients are assigned a mutually exclusive treatment outcome at the end of their therapy, by a health professional, based on the WHO definitions [8]. Specifically, cure was defined as someone who completed treatment without evidence of treatment failure and who had three or more consecutive negative cultures taken at least 30 days apart, after the intensive phase [8]. Treatment completion was defined as someone who completed treatment, without evidence of failure but with no record that three or more consecutive negative cultures taken at least 30 days apart after the intensive phase. Treatment failure was defined as treatment terminated or a need for permanent regimen change of at least two anti-TB drugs due to lack of culture conversion by the end of the intensive phase, or bacteriological reversion in the continuation phase after conversion to negative after the intensive phase, or evidence of additional acquired resistance to fluoroquinolones or second-line injectable drugs. Lost to follow-up was defined as a patient whose treatment was interrupted for two consecutive months or more. Death was defined as someone who died for any reason during the course of treatment. Treatment success was defined as the sum of those who were cured and who completed treatment. A poor treatment outcome was defined as the sum of the treatment outcomes: treatment failure or death.

We calculated the sensitivity and specificity of conversion to predict a successful and poor treatment outcome. In our study, sensitivity refers to the proportion of MDR-TB patients with a successful treatment outcome who had sputum conversion at a given month. Specificity refers to the proportion of MDR-TB patients with a poor treatment outcome who did not have sputum conversion at a given month. We defined positive predictive value (PPV) as the proportion of MDR-TB patients in whom treatment was successful among all those with sputum conversion at a given month. Negative predictive value (NPV) was defined as the proportion of MDR-TB patients in whom treatment outcome was poor among all those with no sputum conversion at a given month. A more detailed definition of variables included in this study is presented in the supplementary information (S1Table).

### Statistical analysis

Time to initial sputum culture and smear conversion was analysed using the Kaplan-Meier estimate and differences between groups were compared using the log-rank test. For patients whose sputum culture or smear result did not convert at all, time to initial conversion was considered to be censored one month before their last sputum specimen date.

We used bivariate and multivariate random-effects logistic regression models to determine the association between sputum conversion and treatment outcome at different months (i.e. at 2, 4, 6, and 12 months), and odds ratios with their 95% confidence intervals (CI) were calculated. Sputum conversion was included in the model as the main independent variable; and demographic variables such as age, sex and occupation, as well as clinical variables such as HIV status, history of previous treatment with second line TB drugs, resistance to a fluoroquinolone or second line injectable TB drugs, and study setting, were included in the adjusted models as cofounding factors. Significance was determined at 5%.

We calculated sensitivity and specificity along with their 95% CIs at different months since commencement of treatment (i.e. 2, 4, 6, and 12 months) using a bivariate random-effects generalized linear mixed model. We preferred this model because it can handle correlations that occur as a result of repeated observations in each individual and it can accommodate heterogeneity between study settings and groups[20]. We calculated coefficients that demonstrated the magnitude and significance of association between each factor and the probability of correct prediction of treatment success (i.e. sensitivity) and poor treatment outcome (i.e. specificity) by conversion status.

Since positive and negative predictive values depends on the prevalence of the events, we assumed that the overall prevalence of MDR-TB treatment success is 50% (based on the MDR-TB treatment success rate reported in WHO’s Global TB Report 2015) [21]. We performed a series of sensitivity analyses for positive and negative predictive values of sputum conversion in which we considered successful treatment outcomes of 40%, 60% and 80% (S2 Table).

We used receiver operating characteristic (ROC) curves to present the sensitivity and specificity of sputum conversion at different months. The Area under the ROC curve (AUC) and Youden's index (i.e. sensitivity + specificity-1) were used to determine the optimal time points of sputum conversion as a marker of a successful MDR-TB treatment outcome. The index closest to one was used as the criterion for determining the best predictor of treatment outcomes. All analyses were performed using STATA version 14.1 [22].

### Ethics clearance

Ethics approval was obtained from the Australian National University Human Research Ethics Committee (protocol number 2016/218) and from the Institutional Review Board of the University of Gondar. Permission was granted to access the secondary data from Tuberculosis Control Institute of Hunan Province and this was documented in a letter. The study was conducted in collaboration with researchers from Gondar University and Hunan Chest Hospital.

## Results

### Demographic and clinical characteristics

A total of 721 patients were diagnosed with MDR- TB in the two hospitals, 490 from Hunan Chest Hospital, China and 231 from Gondar University Hospital, Ethiopia, for the period 2011–2014 for Hunan Chest Hospital and 2010–2014 for Gondar University Hospital. Of these, 120 were culture or sputum smear negative at the commencement of treatment and 12 were diagnosed with extensively drug resistant (XDR)-TB, and they were excluded from the study. There were an additional 19 cases who were excluded from the study (i.e. 10 patients who were diagnosed with MDR-TB but did not start treatment, and 9 patients whose treatment outcomes were not recorded). From a total of 570 bacteriologically confirmed MDR-TB patients who had a treatment outcome recorded, 92 (16%) were from Gondar University Hospital and 478 (84%) were from Hunan Chest Hospital. More than two thirds, (239; 69%) were male, and 407 (71%) were farmers. The median age was 38 years (range, 15 to 87 years). A total of 539 (94%) patients had a history of previous TB treatment, and 138 (24%) had received second-line TB drugs before the initiation of the current MDR-TB treatment. The baseline demographic and clinical characteristics of the patients are presented in supplementary information (S3 Table).

### Smear and culture conversion and treatment outcomes

**Fig 1:**
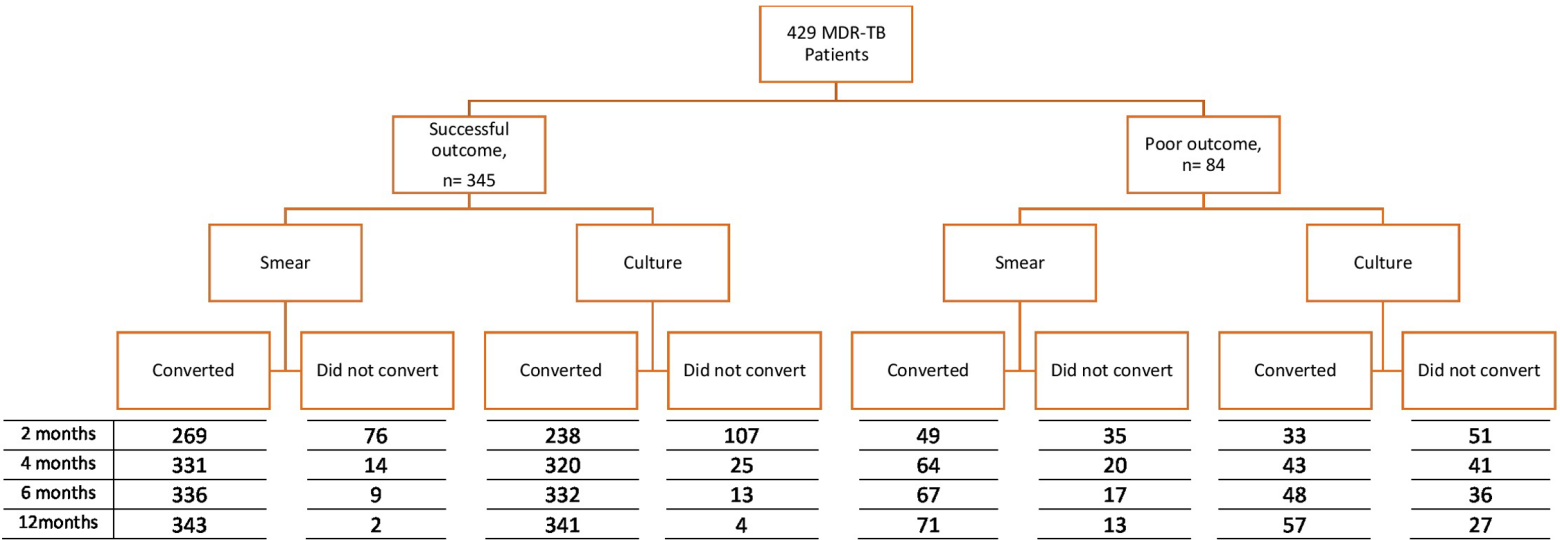
Number of patients with sputum smear and culture conversion after 2, 4, 6, and 12 months of treatment for multidrug resistant tuberculosis, among patients from Hunan Chest and Gondar University Hospitals, 2010 to 2014, stratified by successful and poor treatment outcomes.

Overall, 325 (57%) patients were cured, 20 (3%) patients completed treatment, 18 (3%) died during the treatment, 66 (12%) experienced treatment failure, and 141 (25%) patients were lost to follow-up. After excluding the 141 patients who were lost to follow up, there were 429 patients with MDR-TB. Among this patient group, 345 (80%) had a successful treatment outcome and 84 (20%) had a poor outcome (Fig 1).

**Fig 2:**
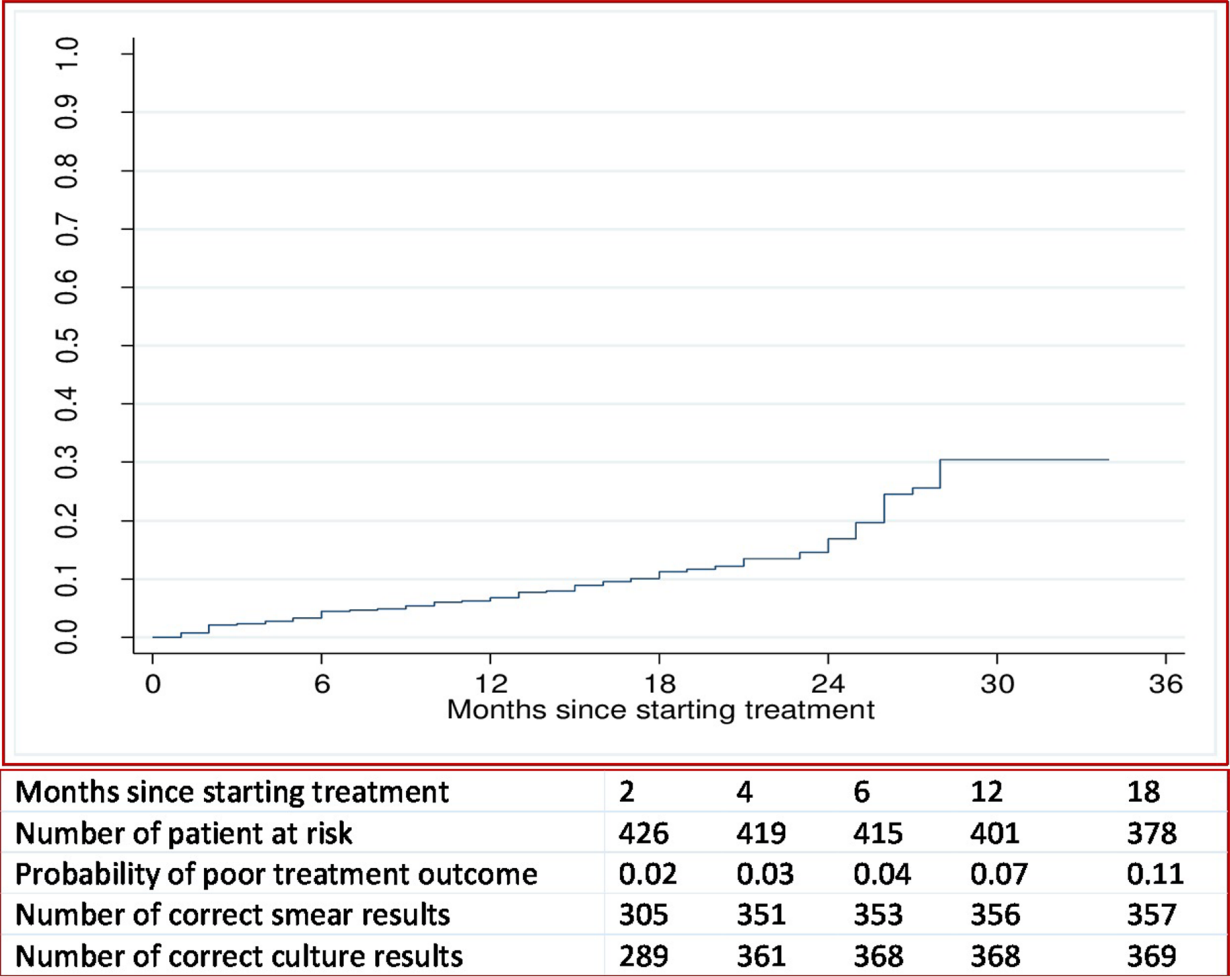
Overall probability of a poor treatment outcome, and number of correct sputum smear and culture results at time-points in patients with multidrug resistant tuberculosis in Hunan Chest and Gondar University Hospital, 2010 to 2014.

The probability of a poor treatment outcome (i.e. death or treatment failure) by the end of 6 months was 4%, by the end of one year was 7%, and by the end of three years was 30% (Fig 2).

**Fig 3:**
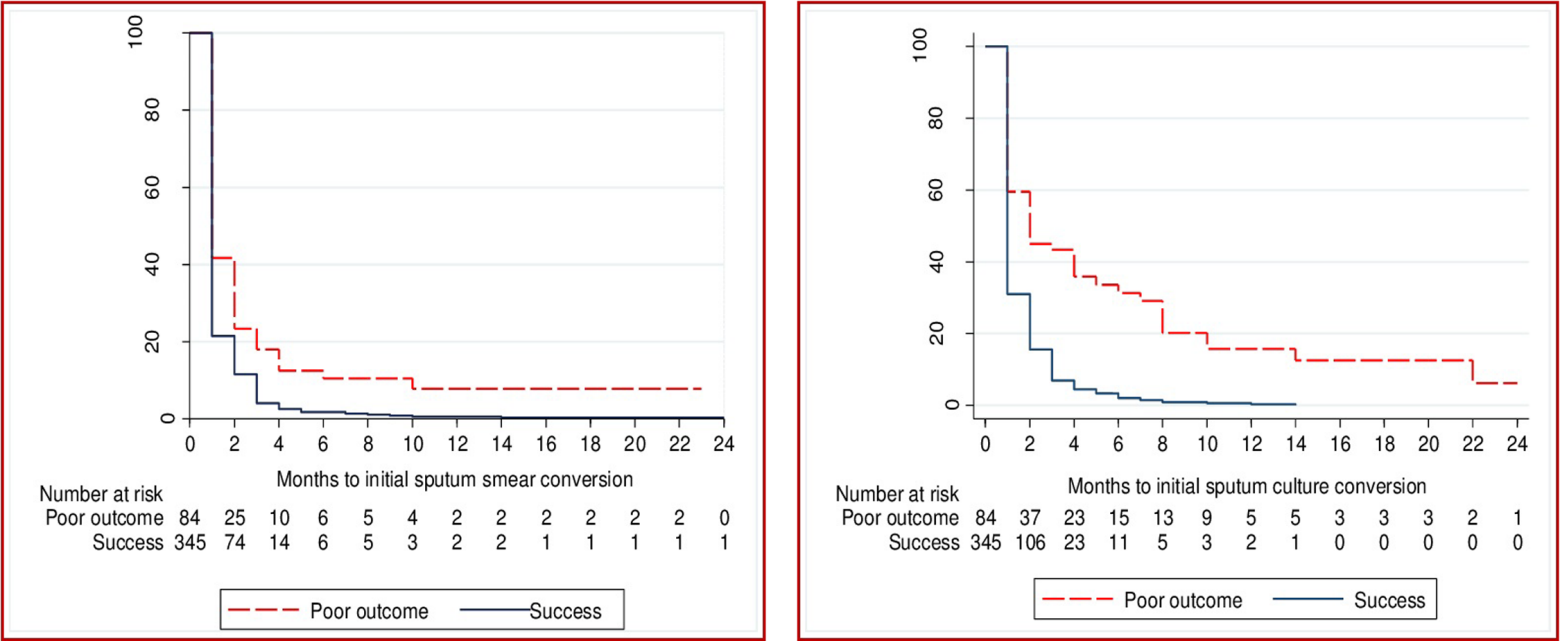
Time to initial sputum smear and culture conversion in patients with multidrug resistant tuberculosis by treatment outcomes from Hunan Chest and Gondar University Hospitals, 2010–2014.

The time to initial culture conversion was shorter in those who had a successful treatment outcome when compared to those who had a poor treatment outcome (p<0.001, Fig 3).

Similarly, the time to initial smear conversion was shorter in those who had a successful treatment outcome compared to patients with a poor treatment outcome (p<0.001, Fig 1). Initial smear conversion occurred for 417 (97%) patients. Of those with a successful treatment outcome, 345 (99%) experienced initial smear conversion; this figure was 84% among those with a poor treatment outcome. A total of 76% of initial smear conversions occurred within two months of treatment commencement. Initial culture conversion occurred in 401 patients (93%); one third (67%) of culture conversions occurred within two months of treatment commencement (Fig 1).

Among those patients who had an initial smear conversion, 75 patients (18%) subsequently had at least one positive smear result (i.e. smear reversion). The median time of smear reversion from the date of treatment commencement was 6.5 months (IQR: 5–12 months). Among patients who had had an initial culture reversion, 72 patients (17%) had culture reversion, at a median time of 8 months (IQR: 5–12 months) after the commencement of treatment.

#### Association between culture and smear conversion, and MDR-TB treatment outcomes

Patients who had initial smear conversion at two months had a higher odds of treatment success compared to those with no smear conversion at two months (crude odds ratio (COR): 2.53; 95%CI: 1.53–4.18) (Table 1). Similarly, patients who had initial culture conversion at two months had a higher odds of treatment success than those without evidence of culture conversion at two months (COR: 3.43; 95%CI: 2.09–5.63). When we compared the strength of association between treatment succuss and culture conversion with that of treatment success and smear conversion at each month in the adjusted models, the odds of treatment success was higher for culture conversion than for smear conversion (Table 2). The strength of association between successful treatment outcome and culture and smear conversion increased with increased months of conversion (Table 2). Having a history of previous, second-line TB treatment and having baseline resistance to a fluoroquinolone were significantly associated with poor treatment outcomes (Table 2).

**Table 1:** Association of sputum smear and culture conversion status at different months with treatment outcome in patients with multidrug resistant tuberculosis from Hunan Chest and Gondar University Hospitals, 2010–2014.

Months on treatment	Sputum smear			Sputum culture		
	Successful outcome	Poor outcome	Crude odds ratio	Successful outcome	Poor outcome	Crude odds ratio
**2 months**						
Converted	269	35	2.53 (1.53, 4.18)	238	33	3.43 (2.09, 5.63)
Did not convert	76	49	1.00	107	52	1.00
**4 months**						
Converted	331	64	7.39 (3.55, 15.38)	320	43	12.20 (6.76, 22.03)
Did not convert	14	20	1.00	25	41	1.00
**6 months**						
Converted	336	67	9.47 (4.05, 22.15)	332	48	19.15 (9.48, 38.67)
Did not convert	9	17	1.00	13	36	1.00
**12 months**						
Converted	343	71	31.4 (6.93, 142.21)	341	57	40.38 (13.62, 119.73)
Did not convert	2	13	1.00	4	27	1.00

Successful outcome: The sum of the treatment outcomes cured and treatment completed. Poor outcome: The sum of the treatment outcomes death and treatment failure.

**Table 2:** Association of initial sputum smear and culture conversion at different months with treatment success in patients with multidrug resistant tuberculosis from Gondar University and Hunan Chest Hospital, 2010–2014, and adjusted odds ration with 95% confidence interval.

	Initial smear conversion				Initial culture conversion			
	2 months	4 months	6 months	12 months	2 months	4 months	6 months	12 months
**2 months**	**3.54 (1.96, 6.38)**	…	…	…	**5.55 (3.06, 10.07)**	…	…	…
**4 months**	…	**9.84 (4.25, 22.83)**	…	…	…	**32.19 (13.36, 77.52)**	…	…
**6 months**	…	…	**13.56 (5.06, 36.30)**	…	…	…	**34.57 (13.79, 86.70)**	….
**12 months**	…	…	…	**37.03(7.75, 176.88)**	…	…	….	**55.56 (17.34, 177.94)**
**Age (in years)**								
< 35	**1.83 (1.03, 3.26)**	1.74 (0.97, 3.14)	1.84 (1.02, 3.33)	1.72 (0.95, 3.10)	1.92 (1.06, 3.49)	1.91 (0.98, 3.69)	1.98 (1.03, 3.81)	1.87 (0.98, 3.56)
> = 35	1.00	1.00	1.00	1.00	1.00	1.00	1.00	1.00
**Male sex**	0.83 (0.46, 1.49)	0.84 (0.46, 1.53)	0.85 0.47, 1.56)	0.87 (0.48, 1.58)	0.81 (0.44, 1.47)	0.83 (0.43, 1.63)	0.86 (0.44, 1.66)	0.86 (0.45, 1.65)
**Occupation**								
Farmer	1.00	1.00	1.00	1.00	1.00	1.00	1.00	1.00
Employed*	1.52 (0.58, 3.99)	1.97 0.70, 5.52)	1.84 (0.66, 5.13)	2.05 (0.72, 5.88)	1.69 (0.63, 4.56)	1.74 (0.58, 5.25)	1.90 (0.64, 5.66)	1.93 (0.63, 5.87)
Other^	0.69 (0.33, 1.44)	0.65 (0.31, 1.36)	0.65 (0.31, 1.35)	0.71 (0.34, 1.49)	0.73 (0.34, 1.54)	0.48 (0.22, 1.07)	0.59 (0.27, 1.33)	0.52 (0.24, 1.12)
**Year of enrolment**								
2010–2011	1.00	1.00	1.00	1.00	1.00	1.00	1.00	1.00
2012	0.73 (0.13, 3.95)	1.45 (0.25, 8.30)	1.69 (0.29, 9.83)	1.18 (0.21, 6.61)	0.54 (0.09, 3.07)	1.15 (0.18, 7.06)	2.01 (0.32, 12.73)	1.55 (0.27, 8.93)
2013	0.83 (0.15, 4.62)	1.39 (0.24, 8.09)	1.61 (0.27, 9.39)	2.25 (0.23, 7.62)	0.89 (0.15, 5.15)	1.83 (0.28,11.71)	2.89 (0.44, 18.94)	2.28 (0.38, 13.59)
2014	1.40 (0.24, 8.02)	2.24 (0.37, 13.31)	2.75 (0.46, 16.46)	2.25 (0.38, 13.13)	1.32 (0.22, 7.83)	2.41 (0.37, 15.55)	4.58 (0.68, 30.72)	3.12 (0.52, 18.93)
**Enrolled in Gondar University Hospital**	2.37 (0.76, 7.39)	2.11 (0.66, 6.71)	2.15 0.67, 6.90)	1.27 (0.42, 3.85)	3.06 (0.99, 9.44)	9.47 (2.44, 36.72)	4.94 (1.26, 19.29)	1.51 (0.46, 4.98)
**HIV positive**	0.83 (0.14, 4.94)	0.60 (0.09, 3.92)	0.71 (0.10, 4.73)	1.28 (0.17, 9.40)	1.06 (0.17, 6.29)	0.41 (0.05, 3.08)	0.43 (0.05, 3.39)	1.07 (0.13, 8.56)
**History of previous second-line TB treatment**	**0.48 (0.27, 0.85)**	**0.48 (0.27, 0.87)**	**0.49 (0.28, 0.88)**	**0.50 0.27, 0.90)**	**0.54 (0.30, 0.98)**	0.63 (0.33, 1.23)	0.69 0.36, 1.34)	0.55 (0.29, 1.05)
**Resistance to ethambutol**	0.71 (0.41, 1.23)	0.81 (0.46, 1.43)	0.78 (0.44, 1.37)	0.75 (0.42, 1.34)	0.85 (0.48, 1.50)	0.89 (0.46, 1.68)	0.96 (0.51, 1.82)	0.91 (0.49, 1.72)
**Resistance to fluoroquinolones**	**0.32 (0.14, 0.73)**	**0.22 (0.09, 0.49)**	**0.24 (0.11, 0.52)**	**0.20 (0.09, 0.45)**	**0.36 (0.16, 0.83)**	**0.27 (0.10, 0.67)**	**0.24 (0.09, 0.59)**	**0.17 (0.08, 0.41)**
**Resistance to any 2**^**nd**^ **line injectable drugs**	0.88 (0.50, 1.55)	0.92 (0.51, 1.65)	0.88 (0.49, 1.58)	0.93 (0.518, 1.68)	0.85 (0.48, 1.53)	0.99 (0.52, 1.90)	0.87 (0.45, 1.65)	0.82 (0.43, 1.56)

*Employed includes private and government employees as well as daily labourers;

^other incudes people who are unemployed and unknown occupations; the reference group for sputum conversion at each months is “did not convers sputum”

#### Validity of culture and smear conversion

For the prediction of a successful treatment outcome, the overall sensitivity of smear conversion at two months was 77.9% (95%CI: 73.3–82.6) with a statistically significant difference (i.e. p <0.05) across the two study settings (86.8% in Hunan Chest Hospital vs 46.6% in Gondar University Hospital), age group (72.7% among patients aged less than 35 years vs 82.5% in people aged 35 years and above), history of previous, second-line TB-treatment (86.6% with history of previous, second-line TB treatment vs 75.3% with no history of previous, second-line TB treatment), and resistance to any injectable second-line TB drug (82.5% for baseline resistance to any second-line injectable TB drug vs 72.4% with no baseline resistance to any second line injectable TB drug).

The overall sensitivity of smear conversion at six months was 97.3% (95%CI: 95.5–99.2), with significant variation across the two hospitals only (p<0.05; Table 3). The overall sensitivity of culture conversion at two months was 68.9% (95%CI: 64.3–73.6), with significant variation across the two hospitals, age, occupation, year of enrolment, HIV status, and baseline resistance to any second-line injectable TB drug (Table 3). For example, among HIV-positive patients with MDR-TB, the sensitivity of culture conversion at two months was 23.5%, as compared with 71.3% among HIV-negative patients (p < 0.001). In contrast, there was no significant difference in the sensitivity of smear conversion at any time-point when comparing HIV-positive and HIV-negative patients (p >0.5, Table 3). The overall sensitivity of culture conversion (77.9%) and smear conversion (68.9%) at two months was significantly different (p = 0.007). However, the overall sensitivities of culture conversion and smear conversion at four months were not significantly different (p>0.05; Table 3).

**Table 3:** The validity (i.e. sensitivity, specificity) of smear and culture conversion at different months as prognostic markers for treatment outcomes in patients with multidrug resistant tuberculosis from Hunan Chest and Gondar University Hospitals, 2010–2014.

	Initial sputum smear conversion						Initial sputum culture conversion					
	2 months		4 months		6 months		2 months		4 months		6 months	
	Sensitivity % (95% CI)	Specificity % (95% CI)	Sensitivity % (95% CI)	Specificity % (95% CI)	Sensitivity % (95% CI)	Specificity % (95% CI)	Sensitivity % (95% CI)	Specificity % (95% CI)	Sensitivity % (95% CI)	Specificity % (95% CI)	Sensitivity % (95% CI)	Specificity % (95% CI)
**Overall**	77.9 (73.3–82.6)	41.6 (31.1–29.8)	95.9 (93.5–98.3)	23.8 (13.8–33.7)	97.4 (95.5–99.2)	20.2 (10.5–25.6)	68.9 (64.3–73.6)	60.7 (50.2–71.2)	92.7 (90.3–95.1)	48.8 (38.8–58.7)	96.2 (94.3–98.0)	42.8 (33.2–52.4)
**Study area**												
China	86.8 (82.1–91.2)*	38.1(27.3–48.9)	97.0(94.6–99.3)*	21.0(11.6–30.4)	98.5 (96.6–100)*	18.4(9.4–27.3)	80.7(75.9–85.5)**	56.5(45.9–67.1)	97.7(94.9–100)**	46.0(34.9–57.1)	98.1(95.9–100)**	40.7(29.7–51.8)
Ethiopia	46.6 (38.0–55.2)	75.0 (41.6–100)*	92.0(87.5–96.4)	50.0(21.0–78.9)	93.3 (89.7–96.9)	37.5(9.9–65.0)	26.6(17.4–35.8)	100 (67.3–100)*	74.6(69.2–80.1)	75.0(40.8–100)	89.3(85.1–93.5)	62.5(28.4–96.5)
**Sex**												
Male	76.7 (71.3–82.1)	40.9(28.6–53.3)	95.6 (93.0–98.1)	26.2(15.5–36.8)	97.3 (95.2–99.4)	22.9(12.9–32.9)	67.9(61.9–73.9)	55.7(43.6–67.8)	92.5(89.1–95.9)	47.5(35.0–60.0)	96.4(94.0–98.9)	44.2(31.8–56.6)
Female	80.3 (72.8–87.8)	43.4(23.3–63.6)	96.5 (93.0–100)	17.3(0.5–34.3)	97.4(94.5–100)	13.0(3.2–29.3)	70.9(62.5–79.3)	73.9(54.2–93.5)	93.1(88.4–97.8)	52.1(31.7–72.5)	95.7(92.2–99.1)	39.1(18.9–59.3)
**Age (in years)**												
<35	72.8(66.5–72.1)*	36.0(16.7–55.2)	96.9(93.8–99.9)	24.0(7.3–40.6)	97.5(95.0–99.9)	20.0(4.2–35.7)	62.3(55.2–69.4)	64.0(44.8–83.1)	89.5(85.5–93.4)*	48.0(28.4–67.5)	95.0(92.1–97.9)	44.0(24.6–63.3)
> = 35	82.5 (76.5–88.4)	44.0(31.5–55.6)	95.0(92.2–97.9)	23.7(12.8–34.5)	97.2(94.9–99.5)	20.3(10.0–30.5)	74.8(68.2–81.5)*	59.3(46.8–71.7)	95.6(91.8–99.3)	49.1(36.3–61.9)	97.2(94.5–100)	42.3(29.7–54.9)
**Occupation**												
Farmer ^Ƨ^	82.4 (77.1–87.6)	40.9(28.7–53.2)	96.1 (93.6–98.6)	22.9(12.8–33.0)	97.4(95.3–99.4)	19.6(9.9–29.3)	75.9(70.2–81.6)	60.6(48.6–72.6)	93.5(90.2–96.8)	49.1(37.2–61.1)	96.9(94.5–99.4)	44.2(32.3–56.1)
Employed	69.6 (44.9–77.3)*	61.9(14.3–100)	95.0 (87.0–100)	62.9(22.7–99.1)*	96.7 (90.1–95.6)	49.9 (11.7–88.2)*	42.6(24.3–60.9)**	87.4 (40.3–100)*	88.5(77.9–98.2)	87.4(39.4–100)*	95.0(87.2–100)	74.9(28.0–100)
Others	78.4 (60.9–95.9)	33.3(8.6–58.0)	96.0(90.6–100)	6.6(1.3–27.0)	98.0(93.6–100)	6.6(1.2–26.2)	68.6(56.4–80.8)	46.6 (22.4–70.8)	94.1(87.0–100)	26.6(2.5–50.7)	94.1(88.9–99.3)	20(0.4–44.0)
**HIV status**												
Negative	78.6(74.1–83.1)	40.2(29.7–50.7)	95.7(93.5–97.8)	23.1(13.9–32.2)	97.2 (95.5–98.9)	19.5(10.8–28.1)	71.3(66.4–76.2)**	59.7(49.2–70.2)	93.3(90.4–96.0)	48.7(39.9–59.5)	96.3(94.2–98.4)	42.6(31.9–53.3)
Positive	64.7(45.0–84.3)	100(32.8–100)	100 (90.6–100)	50.0 (8.5–100)	100 (92.4–100)	50.0(5.3–100)	23.5(20.0–44.9)	100 (32.8–100)	82.3(70.0–94.2)	50.0(19.0–100)	94.1(85.0–100)	50.0(18.5–100)
**History of previous treatment**												
Yes	77.5(73.0–82.0)	42.1(31.6–52.7)	95.6 (93.5–97.8)	24.0(14.95–33.2)	97.2(95.4–98.9)	20.4(11.8–29.1)	68.3(63.3–73.3)	60.2(49.7–70.7)	92.3(89.4–95.1)	49.3(38.7–60.0)	96.0(93.9–98.0)	43.3(32.7–53.9)
No	85.0(66.8–100)	0.005(0.01–96.2)	100(91.3–100)	0.01 (0.008–83.3)	100(93.0–100)	0.06(0.007 78.6)	80.0(59.7–100)	100 (4.6–100))	100(88.6–100)	0.08(0.009–97.4)	100(91.6–100)	0.9(0.009–96.5)
**History of previous second line TB-treatment**												
Yes	86.6 (78.2–97.1)	41.1(24.6–57.7)	95.8(91.3–100)	20.5(6.2–34.8)	100(93.0–100)	20.5(7.0–34.0)	79.4(68.9–89.9)	73.5(57.5–89.5)*	95.8(89.9–100)	55.8(39.1–72.5)	95.8(91.5–100)	52.9(36.5–69.3)
No	75.3(70.4–80.2)*	42.0(28.3–55.6)	95.9(93.6–98.3)	26.0(14.2–37.7)	98.6(94.9–100)	20.0(8.8–31.1)	66.1(60.7–71.6)	52.0(38.7–30.7)	91.9(88.8–94.9)	44.0(30.2–57.7)	96.3(94.0–98.5)	36.0(22.4–49.5)
**Resistance to a second line injectable drug**												
Yes	82.5 (76.6–88.4)*	42.5(29.4–55.7)	95.7 (92.9–98.5)	22.2(10.8–33.5)	97.8(95.6–100)	18.5(7.8–29.2)	74.6(68.0–81.1)*	61.1(48.0–74.1)	95.2(91.5–98.9)	50.0(36.6–63.3)	98.4(95.7–100)	44.4(31.2–57.6)
No	72.4 (65.9–78.8)	40.0(22.3–57.6)	96.1 (93.0–99.2)	26.6(11.4–41.8)	96.7(94.2–99.2)	23.3(8.9–37.6)	62.1(59.9–69.3)	60.0(42.5–77.4)	89.7(85.6–93.7)	46.6 (28.7–64.5)	93.5(90.6–96.5)	40.0(22.3–57.6)
**Resistance to ethambutol**												
Yes	76.4(68.5–84.3)	33.3(16.6–49.9)	92.4(88.7–96.1)*	24.2(9.7–38.7)	95.2(92.2–98.3)	18.1(4.4–31.8)	65.0(56.3–73.8)	63.6(46.9–80.2)	91.5(86.5–96.4)	54.5(37.5–71.5)	95.2(91.6–98.9)	51.5(34.7–68.2)
No	78.6 (73.4–83.9)	47.0(33.6–60.4)	97.4 (95.0–99.9)	23.5(11.8–35.2)	98.3(96.3–100)	21.5(10.5–32.5)	70.7(64.8–76.5)	58.8(45.4–72.2)	93.3(90.0–96.5)	45.0(31.4–58.7)	96.6(94.2–99.0)	37.2(23.8–50.7)
**Resistance to a fluoroquinolone**												
Yes	76.4(58.7–96.1)	52.9(29.6–76.2)	100(90.6–100)	11.7(0.8–31.8)	100(92.4–100)	11.7(7.2–30.5)	58.8(36.8–80.7)	64.7(41.5–87.9)	94.1(81.7–100)	47.0(23.3–70.8)	94.1(85.0–100)	35.2(11.8–58.7)
No	78.0(73.5–82.5)	38.8(27.0–50.5)	95.7 (93.5–97.8)	26.8 (16.7–26.9)	97.2(95.5–98.9)	22.3(12.8–31.95)	69.5(64.5–74.5)	59.7(48.0–71.3)	92.6(89.8–95.4)	49.2(37.2–61.2)	96.3(94.2–98.4)	44.7(32.9–56.5)

*P-value < 0.05;

**p-value<0.001;

^Ƨ^ Reference group

For the prediction of a poor treatment outcome, the specificity of sputum smear conversion was significantly lower than culture conversion at all time-points (i.e. 41.6% vs 60.7% at 2 months; 23.8% vs 48.8% at four months; 20.2% vs 42.8% at six months; and 15.4% vs 32.1% at 12 months; Table 3). The specificity of culture conversion at two months varied by study setting (i.e. hospitals) and by history of previous treatment with second-line TB drugs; whereas the specificity of sputum smear conversion at two months varied only by study setting (Table 4).

**Table 4:** The validity of sputum smear and culture conversion at different months as prognostic markers for treatment success in patients with multidrug resistant tuberculosis, from Hunan Chest and Gondar University Hospitals, 2010–2014.

	Sputum smear conversion % (95% CI)	Sputum culture conversion % (95% CI)	P-value
**2 months**			
Sensitivity	77.9 (73.6–82.3)	68.9 (64.1–73.8)	0.007
Specificity	41.6 (31.1–52.2)	60.7 (50.3–71.1)	0.012
Area under ROC* curve	0.60 (0.54–0.65)	0.64 (0.59–0.71)	0.086
Positive likelihood ratio	1.34 (1.11–1.62)	1.76 (1.33–2.31)	N/A
Negative likelihood ratio	0.52 (0.38–0.72)	0.51 (0.40–0.64)	N/A
Positive predictive value	57.3 (52.6, 61.8)	63.7 (57.1–69.8)	N/A
Negative predictive value	65.7 (58.1, 72.6)	66.2 (60.8–71.2)	N/A
Youden-index	19.5	29.6	NA
**4 months**			
Sensitivity	95.9 (93.8–98.0)	92.7 (90.0–95.5)	0.069
Specificity	23.8 (14.7–32.9)	48.8 (38.1–59.5)	< 0.0001
Area under ROC* curve	0.60 (0.55–0.64)	0.71 (0.65–0.76)	< 0.0001
Positive likelihood ratio	1.26 (1.12–1.42)	1.81(1.47–2.24)	N/A
Negative likelihood ratio	0.17 (0.09–0.32)	0.15(0.10–0.23)	N/A
Positive predictive value	55.7 (52.7–58.7)	64.4 (59.5–69.1)	N/A
Negative predictive value	85.4 (75.6–91.8)	87.1 81.3–91.2)	N/A
Youden-index	19.7	41.5	NA
**6 months**			
Sensitivity	97.4 (95.7–99.0)	96.2 (94.2–98.2)	0.386
Specificity	20.2 (11.6–28.8)	42.8 (32.2–53.4)	0.001
Area under ROC* curve	0.59 (0.54–0.63)	0.69 (0.64–0.75)	<0.0001
Positive likelihood ratio	1.22(1.09–1.36)	1.68 (1.40–2.03)	N/A
Negative likelihood ratio	0.13(0.06–0.28)	0.09 (0.05–0.16)	N/A
Positive predictive value	55.0 (52.3–57.7)	62.7 58.3–67.0)	N/A
Negative predictive value	88.6 (78.2–94.4)	91.9 86.3–95.3)	N/A
Youden-index	17.6	39	NA
**12 months**			
Sensitivity	99.4 98.6–99.9)	98.9 (97.7–99.7)	0.412
Specificity	15.4 (7.7–23.2)	32.1 (22.1–42.1)	0.01
Area under ROC* curve	0.57 (0.53–0.61)	0.65 (0.60–0.70)	0.0001
Positive likelihood ratio	1.18 (1.07–1.29)	1.46 (1.26–1.69)	N/A
Negative likelihood ratio	0.04 (0.01–0.16)	0.04 (0.01–0.10)	N/A
Positive predictive value	54.0 (51.8–56.3)	59.3 (55.7–62.8)	N/A
Negative predictive value	96.4 (86.0–99.1)	96.5 90.9–98.7)	N/A
Youden-index	14.8	30.9	NA

*ROC: Receiver operating characteristic;

N/A: Not Applicable. To calculate the positive and negative predictive values we assumed that the overall prevalence of MDR-TB treatment success is 50%.

### Positive and negative predictive values

The positive and negative predictive values for smear conversion were: 57.3% and 65.7% at two months, 55.7% and 85.4% at four months, and 55.0% and 88.6% at six months; and for culture conversions it was: 63.7% and 66.2% at two months, 64.4% and 87.1% at four months, and 62.7% and 91.9% at six months, respectively (Table 4).

If the patient converted their smear from positive to negative by two months, the probability of a successful treatment outcome was 57.3%. If a patient converted from culture positive to negative by two months, the probability of a successful treatment outcome was 63.7%. The positive predictive values of culture conversion were higher than the positive predictive values of sputum smear conversion at all time-points (S2 Table). The negative predictive values of sputum smear conversion were similar to culture conversion at all time-points (Table 4).

### Optimal time of culture and sputum smear conversion

**Fig 4:**
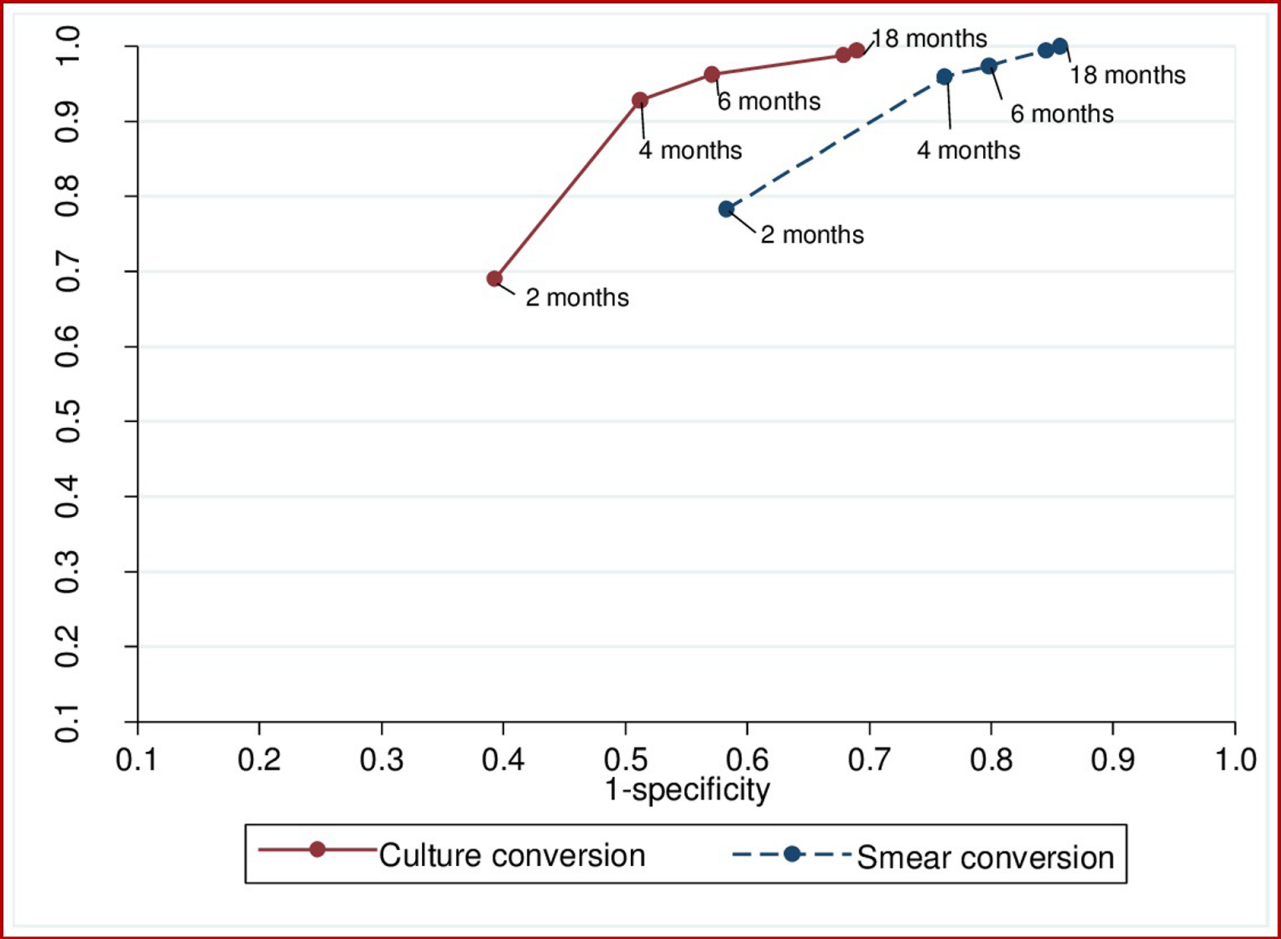
The performance of smear and culture conversion at different months after commencement of treatment for multi-drug resistant tuberculosis to predict treatment outcome (successful vs poor treatment outcomes), for patients from Hunan Chest and Gondar University Hospitals, 2010 to 2014.

The sensitivity of both culture and sputum smear conversion increased over time, whereas specificity decreased (Fig 2). The combined sensitivity and specificity to predict a successful treatment outcome peaked between two and four months for sputum smear conversion, and between four and six months for culture conversion (Fig 4).

The overall prognostic performance of culture conversion at two months in predicting successful MDR-TB treatment outcomes was not significantly differ to that of sputum smear conversion at two months (p = 0.86). However, the prognostic performance of culture conversion at four, six, and 12 months was significantly higher than the performance of sputum smear conversion at the respective months (p<0.05; Table 3). The common optimum time point for sputum smear and culture conversion was 4 months; at this time-point, culture conversion (AU_ROC_ curve = 0.71) was significantly better than sputum smear conversion (AU_ROC_ curve = 0.6) in predicting successful MDR-TB treatment outcomes (p< 0.001).

**Fig 5:**
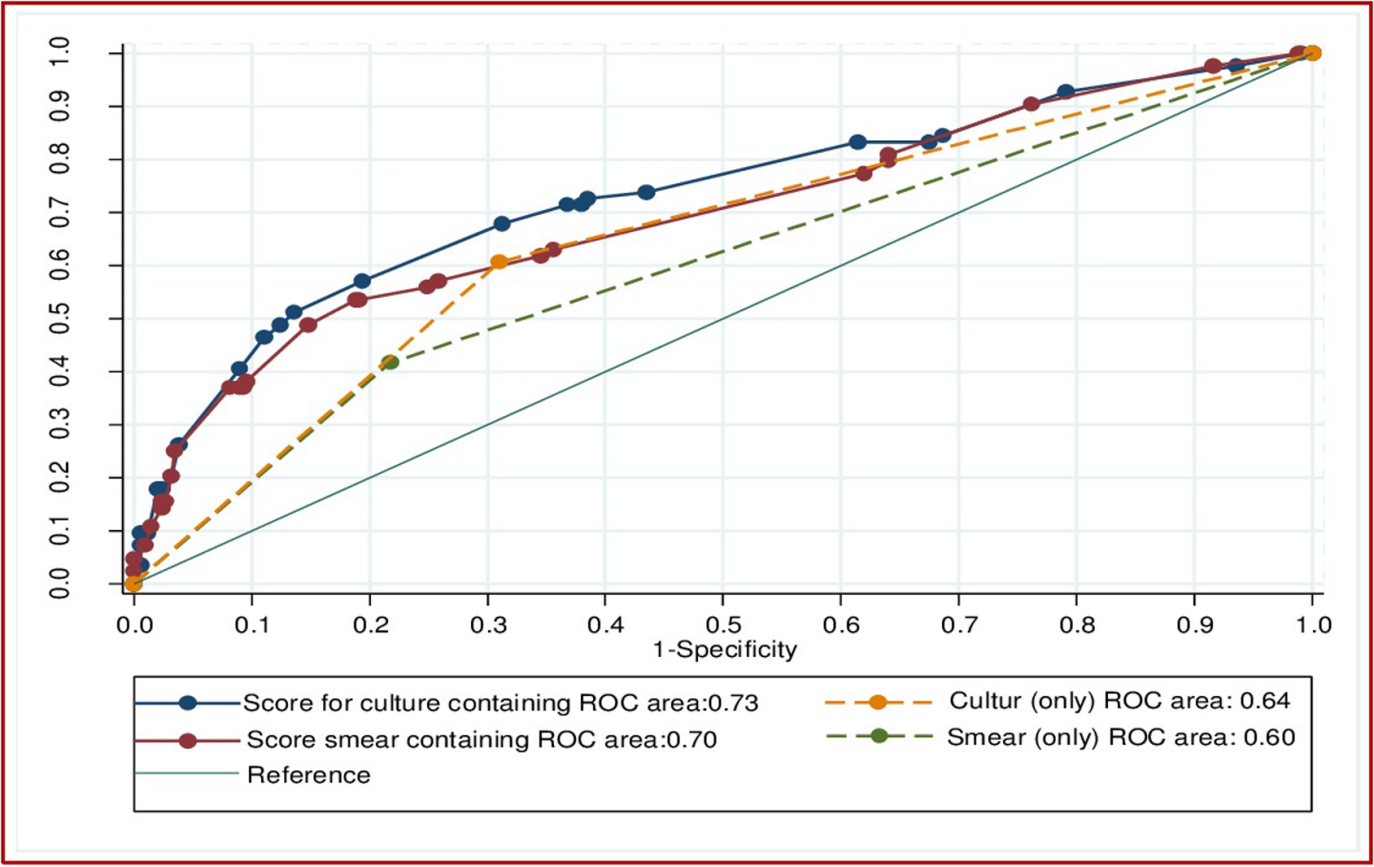
Discriminatory performance of risk scores for differentiating poor treatment outcomes from successful treatment outcomes among patients with multi drug resistant tuberculosis, using smear and culture conversion at two months only, as well as other demographic and clinical factors, from Hunan Chest and Gondar University Hospitals, 2010–2014.

Fig 5 showed the AU_ROC_ curve for differentiating poor treatment outcomes using smear and culture conversion at two months, with and without the inclusion of other demographic and clinical factors. The AU_ROC_ curve increased from 0.6 for smear conversion (only) to 0.7 when other significant variables (i.e. age of the patient, resistance to fluoroquinolones, a history of second-line, injectable TB treatment) were included in the score. Similarly, the AU_ROC_ curve for culture conversion (AU_ROC_ curve = 0.64) increased when significant variables were included in the model (AU_ROC_ curve = 0.73) (Fig 5).

## Discussion

We assessed and compared the validity and predictive value of sputum smear and culture conversion at different months since treatment commenced as prognostic markers for end-of-treatment outcomes among MDR-TB patients from Hunan Chest Hospital, China and Gondar University Hospital, Ethiopia. Our study demonstrated that the sensitivity of culture and smear conversion increased as the month of conversion to negative increased, but at the cost of decreased specificity. The optimum time points to predict a future successful treatment outcome were between two and four months after treatment commencement for sputum smear conversion and between four and six months for culture conversion. Culture conversion was significantly better than smear conversion in predicting final treatment outcomes. The prognostic performances of both sputum culture and smear conversion were improved when the demographic and clinical risk factors were included in the risk score.

We found that culture conversion (69%) and sputum smear conversion (78%) at two months have poor sensitivity for predicting successful treatment outcomes. This is consistent with findings of previous studies [16, 17]. The reason for poor sensitivity of culture and sputum smear conversion at two months may be due to the fact that many of the patients who remain culture or sputum smear positive at two months later convert to negative and have successful treatment outcomes. In our study, among those patients who had demonstrated sputum conversion, 75 (18%) had smear reversion, and of these 72 (17%) reverted back to being negative. The other reason could be that sputum smear and culture conversion at two months were significantly affected by clinical and socio-demographic factors. For example, sputum smear and culture conversion at two months were significantly lower among younger patients, in patients with a history of previous second-line TB-treatment, and in patients with resistance to second line injectable TB drugs.

The validity (i.e. sensitivity and specificity) of two-month sputum smear and culture conversion were not high enough to predict final MDR-TB treatment outcomes. Other demographic and clinical factors played significant roles in predicting treatment response, and influenced the validity of culture and sputum smear conversion at two months post-treatment. In previous studies, demographic and clinical factors have also been reported as strong predictors of treatment response [15, 23, 24]. This suggests the importance of developing composite prognostic markers that can predict end-of-treatment outcomes in the early stage of MDR-TB treatment that may have the potential to guide clinical decision making.

On the other hand, the sensitivity of six-month sputum smear and culture conversion was high (97% for sputum smear and 96% for culture conversion), but the specificity was very low (20% for sputum smear and 43% for culture conversion). The reason for the low specificity is that many initial sputum smear and cultures conversions subsequently reverted. Another study reported similar findings, i.e. that sensitivity at six months was high but with modest specificity [16], and late culture conversion was associated with poor treatment outcomes [25, 26].

The optimum combined sensitivity and specificity for culture conversion occurred between month four and month six of treatment, and for sputum smear conversion it occurred between month two and month four of treatment. The common optimum times for culture and smear conversion together was four months. At this month, culture conversion was significantly better than sputum smear conversion in predicting the final treatment outcomes of the patients, suggesting the importance of using both sputum smear and culture examination rather than sputum smear alone for monitoring of MDR-TB patients. The current WHO treatment guideline for MDR-TB recommends the use of sputum smear microscopy and culture together rather than sputum smear microscopy alone for the monitoring of patients with MDR-TB, throughout treatment[5]. Our results support this recommendation.

The combined sensitivity and specificity of culture and smear conversion at two months were improved when the demographic (i.e. age of the patient) and clinical risk factors (i.e. resistant to fluoroquinolones, history of second-line injectable TB treatment) were included in the risk score. When the clinical and demographic variables were included in the score, the AU_ROC_ curve of the two month smear conversion (0.7) was almost equal to the AU_ROC_ curve of culture conversion at four months (0.71). This indicated that developing a comprehensive clinical risk score and validating the score in a prospective cohort study will improve the prognostic capacity of smear and culture conversion in patients with MDR-TB.

Predictive values were used to estimate the probability that the outcome will occur on a sputum conversion. We found high NPVs for sputum smear and culture conversion after four months of treatment. This indicates that if a patient did not convert to sputum smear and culture negative by four months, the probability of a poor treatment outcome was high (85% - 87%). On the other hand, we found relatively high PPVs for culture conversion at four months, compared to smear conversion at four months. This implies that the patients will have a high probability of treatment success if they have experienced culture conversion at four months. However, sputum smear conversion at four months is not a good prognostic marker of a successful treatment outcome.

The notable limitation associated with the present study is its retrospective design and inability to identify the actual days of culture and smear conversion, as sputum examinations were carried out at a certain month after treatment commencement. The other limitation of the study is that it might not be generalizable to patients with MDR-TB who are enrolled in other MDR-TB programs. We observed that there are differences in the validity of culture and sputum smear conversion between the two settings, suggesting that our results may not be generalizable. For example, the sensitivity of smear conversion, for the prediction of a successful treatment outcome, at two months was significantly higher in Hunan Chest Hospital (86.8%) than in Gondar University Hospital (46.6%) (i.e. p <0.05). Thus, the validity of sputum smear and culture conversion to predict treatment outcomes might be dependent on several factors relating to the study context, including the level of socioeconomic development of the country, health systems factors and or other factors that we were not able to account for in our study [17, 27, 28]. A multicentre prospective cohort study conducted by Kurbatova et al showed that the validity of initial sputum culture conversion to predict MDR-TB treatment outcome varied by country; at two months the sensitivity was 9% in South Africa and 66% in Taiwan, and the specificity was 94% in South Africa and 75% Taiwan [28]. Another study conducted in urban settings in Jiangsu province, China showed that the sensitivity and specificity of culture conversion were 33.3% and 80.0% at two months, and 90.5% and 56.4% at six months, respectively [17].

## Conclusion

The validity of smear conversion is significantly lower than culture conversion in predicting successful TB treatment outcomes for patients with MDR-TB. We support the WHO recommendation of using both smear and culture examination rather than smear alone for the monitoring of MDR-TB patients for a better prediction of successful treatment outcomes. The optimum time points to predict a future successful treatment outcome were between two and four months after treatment commencement for sputum smear conversion and between four and six months for culture conversion. The common optimum times for culture and smear conversion together was four months. Demographic and clinical factors played significant roles in predicting treatment outcomes, and influenced the validity of culture and smear conversion at two months post-treatment.

## Supporting information

S1 Table Definition of variables which were used in this study. (DOCX)

S2 Table Positive and negative predictive value of initial sputum smear and culture conversion in predicting treatment outcome (successful versus poor treatment outcomes) from Hunan Chest Hospital, China and University of Gondar, Ethiopia: 2010–2014. (DOCX)

S3 Table Baseline demographic and clinical characteristics of patients with multidrug resistant tuberculosis from Hunan Chest Hospital, China and University of Gondar, Ethiopia: 2010–2014. (DOCX)
