# Supplementary material for: Comparison of the validity of smear and culture conversion as a prognostic marker of treatment outcome in patients with multidrug-resistant tuberculosis
Source: PLoS One. 2018 May 23;13(5):e0197880. doi: 10.1371/journal.pone.0197880 (PMC5965863; doi:10.1371/journal.pone.0197880)
Supplement: S1 Table — (DOCX) [file pone.0197880.s001.docx]

**S1 Table:** Definition of variables which were used in this study

| **Variables** | **Definitions** |
| --- | --- |
| Multidrug resistant tuberculosis (MDR-TB) | Tuberculosis resistant to at least isoniazid and rifampicin |
| Extensively drug resistant tuberculosis (XDR-TB) | Tuberculosis resistant to at least isoniazid, rifampicin, any fluoroquinolone and at least one second-line injectable drug |
| Culture conversion | Two consecutive negative sputum cultures taken at least 30 days apart following an initial positive culture |
| Smear conversion | Two consecutive negative sputum smears taken at least 30 days apart following an initial positive smear |
| Time to initial sputum culture conversion | The time in months from the date of start of MDR-TB treatment to the date of specimen collection for the first of the two consecutive negative cultures. |
| Time to initial sputum smear conversion | The time in months from the date of start of MDR-TB treatment to the date of specimen collection for the first of the two consecutive negative smears. |
| Sustained culture conversion | An absence of any subsequent positive cultures after conversion |
| Sustained smear conversion | An absence of any subsequent positive smear after conversion |
| Culture reversion to positive | At least one subsequent positive culture after initial conversion |
| Smear reversion to positive | At least one subsequent positive smear after initial conversion |
| Sputum smear reconversion | At least one subsequent positive smear after initial conversion |
| Persistent culture positivity | No culture conversion in patients with a baseline positive culture |
| Persistent smear positivity | No smear conversion in patients with a baseline positive smear |
| Cured | An MDR-TB patient who completed treatment without evidence of treatment failure and who had three or more consecutive negative cultures taken at least 30 days apart, after the intensive phase |
| Treatment completion | An MDR-TB patient who has completed treatment, without evidence of failure but with no record that three or more consecutive cultures taken at least 30 days apart were negative after the intensive phase |
| Treatment failure | Treatment terminated or a need for permanent regimen change of at least two anti-TB drugs due to an adverse drug reaction, or lack of culture conversion by the end of the intensive phase, or bacteriological reversion in the continuation phase after conversion to negative after intensive phase, or evidence of additional acquired resistance to fluoroquinolones or second-line injectable drugs |
| Lost to follow-up | A patient whose treatment was interrupted for two consecutive months or more |
| Death | An MDR-TB patients who died for any reason during the course of MDR-TB treatment |
| Transfer out | An MDR-TB patient who has been transferred to another reporting and recording unit and for whom the treatment outcome is unknown |
| Treatment success | The sum of the treatment outcomes cured and treatment completion |
| Poor outcome | The sum of the treatment outcomes treatment failure and death |
| Sensitivity | The proportion of patients with sputum culture conversion by month 2 and month 6 among those with successful treatment outcome |
| Specificity | The proportion of patients with no conversion by month 2 and month 6 among those with poor treatment outcome |
| Positive predictive value | The proportion of patients in whom treatment was successful among all those with initial sputum conversion |
| Negative predictive value | The proportion of patients in whom poor treatment outcome occurred among all those with no sputum conversion |
