# Supplementary material for: Comparison of the validity of smear and culture conversion as a prognostic marker of treatment outcome in patients with multidrug-resistant tuberculosis
Source: PLoS One. 2018 May 23;13(5):e0197880. doi: 10.1371/journal.pone.0197880 (PMC5965863; doi:10.1371/journal.pone.0197880)
Supplement: S2 Table — (DOCX) [file pone.0197880.s002.docx]

**S2 Table:** Positive and negative predictive value of initial sputum smear and culture conversion in predicting treatment outcome (successful versus poor treatment outcomes) from Hunan Chest Hospital, China and University of Gondar, Ethiopia: 2010-2014

|  | Positive predictive value based on the proportion of patients with a successful treatment outcome | | | | Negative predictive value based on the proportion of patients with a successful treatment outcome | | | |
| --- | --- | --- | --- | --- | --- | --- | --- | --- |
|  | 40% | 50% | 60 % | 80% | 40% | 50% | 60 % | 80% |
| Sputum smear conversion | | | | | | | | |
| 2 months | 47%  (42 - 51) | 57%  (53-62) | 67%  (62 - 71) | 84%  (82-87) | 74%  (67 - 80) | 66%  (58 -73) | 56%  (48 - 64) | 32%  (25 -39) |
| 4 months | 46%  (43-49) | 56%  (53- 59) | 65%  (63 - 68) | 83%  (82 - 85) | 90%  (82 - 94) | 85%  (76 - 92) | 80%  (67 - 88) | 59%  (44 -74) |
| 6 months | 45%  (42- 48) | 55%  (52- 58) | 65%  (62 -67) | 83%  (81 - 84) | 92%  (84 - 96) | 89%  (78 - 94) | 84%  (70 - 92) | 66%  (47 - 81) |
| 12 months | 44%  (42 - 46) | 54%  (52 - 56) | 64%  (62 - 66) | 82%  (81 - 84) | 98%  (90 - 99) | 96%  (86 - 99) | 95%  (80 - 99) | 87%  (60 - 97) |
| Sputum culture conversion | | | | | | | | |
| 2 months | 54%  (47 - 61) | 64%  (57 - 70) | 73%  (67 - 78) | 88%  (84 - 90) | 75%  (70 - 79) | 66%  (61 -71) | 57%  (51 - 62) | 33%  (28 - 38) |
| 4 months | 55%  (49 - 60) | 64%  (59 - 69) | 73%  (69 - 77) | 88%  (85 - 90) | 91%  (87 -94) | 87%  ( 81 -91) | 82%  (74 - 87) | 63%  (52-72) |
| 6 months | 53%  (48 - 57) | 63%  (58- 67) | 72%  (68 - 75) | 87%  ( 85 - 89) | 94%  (90 - 97 ) | 92%  (86 - 95) | 88%  (81 - 93) | 74%  (61 - 84) |
| 12 months | 49%  (46 - 53) | 59%  (56 - 63) | 69%  (65 - 72) | 85%  (83 - 87) | 98%  (94 - 99) | 96%  (91 - 99) | 95%  (87 - 98) | 87%  (71 - 95) |
