# Supplementary material for: Comparison of the validity of smear and culture conversion as a prognostic marker of treatment outcome in patients with multidrug-resistant tuberculosis
Source: PLoS One. 2018 May 23;13(5):e0197880. doi: 10.1371/journal.pone.0197880 (PMC5965863; doi:10.1371/journal.pone.0197880)
Supplement: S3 Table — (DOCX) [file pone.0197880.s003.docx]

**S3 Table:** Baseline demographic and clinical characteristics of patients with multidrug resistant tuberculosis from Hunan Chest Hospital, China and University of Gondar, Ethiopia: 2010-2014

|  |  | Treatment success | |  | Poor treatment outcome | |  |
| --- | --- | --- | --- | --- | --- | --- | --- |
| Characteristics | Total    N=570  n (%) | Cured  N=325  n (%) | Treatment Completed  N=20  n (%) |  | Death  N=18  n (%) | Treatment failure  N=66  n (%) | Lost to follow up  N=141  n (%) |
| **Age in years**  <35  >=35 | 244 (43)  326 (57) | 158 (49)  167 (51) | 9 (45)  11 (5) |  | 5 (28)  13 (72) | 23 (35)  43 (65) | 49 (35)  92 (65) |
| **Sex**  Male  Female | 396 (69)  174 (31) | 211 (65)  114 (35) | 17 (85)  3 (15) |  | 16 (89)  2 (11) | 45 (68)  21 (32) | 107 (76)  34 (24) |
| **Occupation**  Employee*  Farmer  Unemployed**  Daily labourer  Others  Unknown | 54 (10)  402 (70)  28 (5)  31 (5)  22 (4)  33 (6) | 35 (11)  224 (69)  22 (7)  18 (6)  11 (3)  15 (5) | 6 (30)  9 (45)  0  2 (10)  0  3 (15) |  | 3 (17)  11 (61)  2 (11)  1 (6)  1 (6)  0 | 3 (5)  50 (76)  1 (1)  1 (1)  6 (9)  5 (8) | 7 (5)  108 (77)  3 (2)  9 (6)  4 (3)  10 (7) |
| **Year of enrolment**  2010  2011  2012  2013  2014 | 4 (1)  13 (2)  139 (24)  194 (34)  220 (387) | 2 (1)  10 (3)  80 (25)  107 (33)  126 (39) | 1 (5)  2 (10)  6 (30)  9 (45)  2 (10) |  | 1 (6)  1 (6)  8 (44)  4 (22)  4 (22) | 0  0  19 (28)  31(47)  16 (24) | 0  0  26 (18)  43 (30)  72 (51) |
| **Study setting**  Hunan Chest Hospital, China  Gondar University Hospital, Ethiopia | 478 (84)  92 (16) | 256 (79)  69 (21) | 14 (70)  6 (30) |  | 13 (72)  5 (28) | 63 (95)  3 (5) | 132 (94)  9 (6) |
| **HIV**  Positive  Negative | 21 (4)  549 (96) | 15 (5)  310 (95) | 2 (10)  18 (90) |  | 2 (11)  16 (89) | 0  66 (100) | 2 (1)  139 (99) |
| **History of previous TB treatment**  Yes  No | 537 (94)  33 (6) | 306 (94)  19 (6) | 19 (95)  1 (5) |  | 18 (100)  0 | 65 (98)  1 (2) | 129 (91.5)  12 (8.5) |
| **History of previous TB treatment with second line drugs**  Yes  No | 138 (24)  432 (76) | 70 (22)  255 (78) | 3 (15)  17 (85) |  | 8 (44)  10 (56) | 26 (39)  40 (61) | 31 (22)  110 (78) |
| **Resistance to ethambutol**  Yes  No  Unknown | 184 (32)  374 (66)  12 (2) | 100 (31)  222 (68)  3 (1) | 6 (30)  14 (70)  0 |  | 9 (50)  9 (50)  0 | 24 (36)  40 (61)  2 (3) | 45 (32)  89 (63)  7 (5) |
| **Resistance to an injectable second line TB drug**  Yes  No  Unknown | 306 (54)  252 (44)  12 (2) | 177 (55)  145 (44)  3 (1) | 12 (60)  8 (40)  0 |  | 10 (56)  8 (44)  0 | 42 (64)  22 (33)  2 (3) | 65 (46)  69 (49)  7 (5) |
| **Resistant to a fluoroquinolone**  Yes  No  Unknown | 46 (8)  179 (31)  345 (61) | 17 (5)  113 (35)  195 (60) | 0  12 (60)  8 (40) |  | 2 (11)  6 (33)  10 (56) | 15 (23)  15 (23)  36 (54) | 12 (9)  33 (23)  96 (68) |
| Occupation: Employed are both private and government employed; housewives and students were considered as unemployed, *Government employee or self-employed; ** Housewife or student | | | | | | | |
